# In vitro comparison of the osteogenic capability of human pulp stem cells on alloplastic, allogeneic, and xenogeneic bone scaffolds

**DOI:** 10.1186/s12903-023-02726-4

**Published:** 2023-01-31

**Authors:** Marius Heitzer, Ali Modabber, Xing Zhang, Philipp Winnand, Qun Zhao, Felix Marius Bläsius, Eva Miriam Buhl, Michael Wolf, Sabine Neuss, Frank Hölzle, Frank Hildebrand, Johannes Greven

**Affiliations:** 1 Department of Oral and Maxillofacial Surgery, University Hospital of RWTH Aachen, Pauwelsstraße 30, 52074 Aachen, Germany; 2 Department of Orthopedics, Trauma and Reconstructive Surgery, University Hospital of RWTH Aachen, Pauwelsstraße 30, 52074 Aachen, Germany; 3 Institute of Pathology, University Hospital of RWTH Aachen, Pauwelsstraße 30, 52074 Aachen, Germany; 4 Department of Orthodontics, University Hospital of RWTH Aachen, Pauwelsstraße 30, 52074 Aachen, Germany; 5 Helmholtz Institute for Biomedical Engineering, BioInterface Group, RWTH Aachen University, Pauwelsstraße 20, 52074 Aachen, Germany

**Keywords:** Bone grafts, Biocompatibility, Bone tissue engineering, Stem cell

## Abstract

**Background:**

A rigorous search for alternatives to autogenous bone grafts to avoid invasiveness at the donor site in the treatment of maxillomandibular bone defects. Researchers have used alloplastic, allogeneic, and xenogeneic bone graft substitutes in clinical studies with varying degrees of success, although their in vitro effects on stem cells remain unclear. Dental pulp stem cells (DPSCs) can potentially enhance the bone regeneration of bone graft substitutes. The present in vitro study investigates the osteogenic capability of DPSCs on alloplastic (biphasic calcium phosphate [BCP]), allogeneic (freeze-dried bone allografts [FDBAs]), and xenogeneic (deproteinized bovine bone mineral [DBBM]) bone grafts.

**Methods:**

Human DPSCs were seeded on 0.5 mg/ml, 1 mg/ml, and 2 mg/ml of BCP, FDBA, and DBBM to evaluate the optimal cell growth and cytotoxicity. Scaffolds and cell morphologies were analyzed by scanning electron microscopy (SEM). Calcein AM and cytoskeleton staining were performed to determine cell attachment and proliferation. Alkaline phosphatase (ALP) and osteogenesis-related genes expressions was used to investigate initial osteogenic differentiation.

**Results:**

Cytotoxicity assays showed that most viable DPSCs were present at a scaffold concentration of 0.5 mg/ml. The DPSCs on the DBBM scaffold demonstrated a significantly higher proliferation rate of 214.25 ± 16.17 (*p* < 0.001) cells, enhancing ALP activity level and upregulating of osteogenesis-related genes compared with other two scaffolds.

**Conclusion:**

DBBP scaffold led to extremely high cell viability, but also promoted proliferation, attachment, and enhanced the osteogenic differentiation capacity of DPSCs, which hold great potential for bone regeneration treatment; however, further studies are necessary.

## Background

Stem cells have the regenerative potential to treat various diseases and severe tissue damage [1]. Among the various stem cell types found in adults, mesenchymal stem cells (MSCs) have been shown to have promising therapeutic efficacy [1, 2]. Unlike embryonic stem cells (ESCs), MSCs cannot differentiate into the same number of cell lines [2]. MSCs are associated with fewer ethical problems [3] as well as less tumorigenesis compared to induced pluripotent stem cells (iPSCs) [3, 4]. In addition, MSCs have the ability to colonize, proliferate, and differentiate locally after transplantation and can alleviate tissue dysfunction through their trophic activity and formation of new tissue [5, 6]. Various tissues contain MSCs and thus can be obtained from bone marrow, fat, umbilical cords, and dental pulp, among other sources [2, 5, 7]. Previous research has described that, in contrast to bone marrow–derived MSCs, DPSCs may contain a higher percentage of stem and progenitor cells under certain conditions and thus potentially have higher proliferative and osteogenic potential [7]. Compared to MSCs derived from bone marrow or fat, a less invasive procedure is required to obtain DPSCs, which are obtained from molars [6, 8–10], deciduous teeth [8] and premolars extracted during orthodontic treatment [6]. Furthermore, earlier research has reported that even DPSCs from the inflamed pulp tissue of vital teeth can be used, as these cells retain their multipotency and regenerative potential despite undergoing various inflammatory processes [11]. With preserved vitality, DPSCs can also be obtained from clinically accessible teeth with crown fractures [12], exfoliated deciduous [8], and periodontitis [13], thus providing a potentially large supply of stem cells. In vitro and in vivo models have revealed the robust self-renewal and multipotency capacity of DPSCs for the regeneration of complex tissue damage such as bone defects in animal models [6, 14, 15] and human patients [16, 17]. DPSCs thus could potentially be an easily accessible and large-scale cell source for regenerative bone defect therapies [18].

Alveolar process atrophy and maxillomandibular bone defects are caused by jaw injuries, tumor removal or cyst resection [19] and pose therapeutic problems for practitioners as the missing bone volume has to be increased by augmentative procedures [19, 20]. Currently, bone grafts from human donors (autografts) are generally considered the gold standard for bone regeneration of the jaws. Research has also reported on the disadvantages of autografts, including additional donor-site morbidity and limited available supply, necessitating alternatives [21]. Various bone substitutes have been introduced in regenerative dentistry, including alloplastic and allogeneic grafts and xenogeneic bone scaffolds [22–24]. Because of their excellent potential for bone regeneration, alloplastic bone graft substitutes made from biphasic calcium phosphate (BCP) and natural scaffolds made from freeze-dried bone allografts (FDBAs) and deproteinized bovine bone mineral (DBBM) grafts are widely used in regenerative dentistry [25].

Scaffolds made of BCP are widely used because of their favorable resorption and degradation properties [26]. Nevertheless, research has shown that synthetic materials in particular often fail to meet the requirements necessary for complex bone regeneration of the jaw [27]. In addition to synthetic bone substitutes, xenogeneic bone substitutes are increasingly used as an alternative to autologous bone grafts in augmentation procedures [28]. These materials have proven to be biocompatible and osteoconductive, and they have achieved comparable outcomes to autografts and allogeneic scaffolds [29]. While xenogeneic bone substitutes have a bone matrix based on hydroxyapatite and contain organic components, researchers have described the disadvantages of pathogen transmission and immunological tissue reactions [30]. The organic components thus must be removed by various complex physical and chemical cleaning protocols prior to application [31]. FDBA bone grafts pose a lower risk of infection [23] and, as allografts, they most closely match the properties of human bone and are a very reliable alternative to autogenous bone, with comparable clinical outcomes [32].

Most bone graft substitutes can be used as cell delivery systems in the regenerative treatment of bone defects [33]. The cell transfer properties and treatment success depend on the physical properties of the bone graft substitute, including porosity, pore size [34], and pore interconnectivity [23]. Considering the influence of biomaterials on stem cell interactions, studies of stem cell–scaffold interactions in relation to proliferation and differentiation are essential components in the search for a favorable scaffold [35, 36]. Many in vivo studies have investigated stem cell interactions with bone graft substitutes, and the ability to induce bone growth [14, 36]. To date, however, no in vitro studies have investigated the behavior of DPSCs on the bone scaffolds used in the present study. The aim of this study was thus to determine the favorable amount of scaffolding for stem cell cultivation to be used for augmentative procedures of maxillomandibular bone defects. The optimal amounts of BCP, DBBM, and FDBA were investigated in relation to the proliferation, viability, attachment, and osteogenic differentiation of DPSC in vitro.

## Material and methods

### Bone grafts

Three commercially available particulate bone graft substitutes with a particle size of 0.5–2 mm were used in this study; biphasic calcium phosphate (BCP) (maxresorb®, botiss biomaterials GmbH, Germany) was used as the alloplastic scaffold; freeze-dried bone allografts (FDBAs) (maxgraft®, botiss biomaterials GmbH, Germany) and deproteinized bovine bone mineral (DBBM) (cerabone®, botiss biomaterials GmbH, Germany) were used as the allogeneic and xenogeneic bone grafts, respectively (Table 1).

**Table 1:** Bone grafts applied in this study

Type Bone Substitute	Product	Physical Composition	Manufacturer	Chemical Composition	Granule Size (mm)	Porosity (%)	Average Pore Size (µm)
Alloplastic	Maxresorb®	BCP	Botiss	60% HA, 40% β-TCP	0.5–1	80	200–800
Allogeneice	Maxgraft®	FDBA	Botiss	30% NC, 70% HA	< 2	65–80	600–900
Xenogeneice	Cerabone®	DBBM	Botiss	100% HA	0.5–1	65–80	600–900

*BCP* biphasic calcium phosphate; *DBBM* deproteinized bovine bone mineral; *FDBA* freeze-dried bone allografts; *HA* hydroxyapatite; *β-TCP* β-tricalcium phosphate; *NC* natural collagene

### Isolation and cell culture

The DPSCs used in this experiment were harvested from impacted third molars according to an established protocol [23]. In total, 3 impacted third molars were extracted in 3 healthy adult males (18 years old) in the Oral and Maxillofacial Surgery Department of RWTH Aachen University Hospital. All experimental protocols were established and approved by the Ethics Committee of Medical Faculty of RWTH Aachen University (EK 374/19). All methods were carried out in accordance with the guidelines and regulations of Medical Faculty of RWTH Aachen university. The tooth surfaces were cleaned, and the pulp chamber was opened with sterile dental fissure drills in the area of the cementoenamel junction. The pulp tissue was carefully separated from the crown and root by the use of barbed broaches. Prior to the isolation of DPSCs, 1 ml collagenase type I solutions (12 mg/ml, cat. no. 2357206, Gibco, USA) and 1 ml dispase solutions (16 mg/ml, cat. no. 2154769, Gibco, USA) were added into 2 ml sterile phosphate-buffered saline (PBS) containing 100 mg/ml penicillin and 100 mg/ml streptomycin to form a mixed enzymatic solution. The pulp tissues were minced and transferred into the prepared enzyme solution mixture for digestion for 60 min at 37 °C, during which vortexing was performed every 30 min to help break up tissue. Afterward, large-cell aggregates were removed, and single-cell suspensions were obtained by passing cells through a 70 μM cell strainer. Digestion was then terminated by adding 3 ml of basic minimum essential medium (MEM, cat. no. 12571063, Gibco, USA) containing 10% (v/v) fetal bovine serum (FBS, Gibco, USA), and single-cell suspensions were centrifuged at relative centrifugal force (RCF) of 67 g for 5 min at room temperature. The supernatants were carefully aspirated off, and the pellets were re-suspended in 1 ml cell medium and cultured into a 25 cm^2^ cell culture flask at 37 °C in 5% CO_2_.

The isolated DPSCs were cultured in the basic MEM medium (growth medium) containing 10% (v/v) FBS and 1% (v/v) antibiotic–antimycotic solution in 5% CO_2_ at 37 °C, and the medium was refreshed every two days. In the meantime, DPSCs at passages 3 to 5 were utilized in the experiments that followed. For osteogenic induction, the cell growth medium was replaced with osteogenic differentiation medium supplemented with an additional 0.1 mM ascorbic acid, 100 nM dexamethasone, and 10 mM β-glycerophosphate at 80–90% cell confluence.

### Bone scaffold morphology

The morphology of the bone scaffolds was evaluated through SEM. BCP, FDBA, and DBBM scaffolds were gently washed twice with PBS buffer, fixed with 3% glutaraldehyde for 4 h, and then dehydrated in graded concentrations of ethanol (30%, 50%, 70%, 90%, and 100%) twice. Afterward, scaffolds were dried at 37 °C, according to a previously published protocol [37], sputtered with platinum, and observed under a scanning electron microscope (SEM; XL30 FEG; FEI, Eindhoven, the Netherlands) with an acceleration voltage of 10 kV. The porosity of the surface was analyzed using ImageJ software. (Free Java software was provided by the National Institutes of Health, Bethesda, MD, USA.)

### Bone substitute concentration and cytotoxicity assay of DPSCs

In this step, 100 μl of DPSC cell suspension (5.000 cells/well) was added to a 96-well plate and was then cultured overnight in a humidified incubator until monolayer cultures had formed. Cultured DPSCs were then treated for 24 h with cell medium containing increasing concentrations (0.5, 1, and 2 mg/mL) of bone substitute. Then, 10 μl of CCK-8 solution (cat. no. 96992, Sigma, Steinheim, Germany) was added to each well of a 96-well plate and incubated for 2.5 h in darkness, and finally the absorbance was measured at 450 nm using a microplate reader (Infinite M200, Tecan, Switzerland), according to the user’s manual. The results of cell viability were calculated using this formula: Cell Viability (%) = [(OD _experiment group_ − OD _blank group_)/ (OD _control group_ − OD _blank group_)] × 100% (OD stands for absorbance value).

### Cell morphology assessment

After culturing for 7 days, the cellular morphologies on the bone scaffolds at an initial density of 20.000 cells/dish were evaluated through SEM. Cell-scaffold constructs in various groups were gently washed twice with PBS buffer, fixed with 2.5% glutaraldehyde for 4 h, and dehydrated in graded concentrations of ethanol (30%, 50%, 70%, 90%, and 100%) twice. Afterward, cell/scaffold constructs were dried at 37 °C, according to a previously published protocol, [37] sputtered with Au/Pd, and observed under SEM (XL30 FEG; FEI, Eindhoven, the Netherlands) with an acceleration voltage of 10 kV. The experiment was repeated three times and each value collected was analyzed.

### Cell proliferation and attachment on bone graft scaffolds

The sterilized scaffolds were next placed in confocal dishes, and DPSC cells were seeded on BCP, FDBA, and DBBM bone scaffolds at an initial density of 20.000 cells/dish for 3 or 7 days. DPSCs culture without bone substitute served as control. At each predetermined time point, the medium was discarded, the cells were washed three times, and they were then subjected to two different stainings, according to the manufacturer’s instructions: (1) calcein acetoxymethyl ester (calcein AM) staining (live cell staining) (cat. no. PK-CA707-30002, PromoKine, Germany); (2) 4′,6-diamidin-2-phenylindol (DAPI) staining (cat. no. 2381700, Invitrogen, Invitrogen GmbH, Karlsruhe, Germany). Finally, DPSC cells grown on the bone scaffolds were rinsed three times and observed via confocal laser scanning microscopy (CLSM) to determine the proliferative effect. For each group, 8 areas were evaluated at each time point blinded to the source.

Rhodamine phalloidin staining was used to indicate the cytoskeleton of proliferated cells on the bone substitutes after 7 days of cultivation. Therefore, the DPSCs on the bone grafts were first washed three times and fixed in 4% paraformaldehyde for 20 min, followed by pretreatment with Triton X-100 (0.1% v/v) for 15 min. Cell samples were then stained with rhodamine phalloidin (Cat. No. R415, Thermo Fisher, US) for 45 min and DAPI for 5 min. The staining was visualized using CLSM.

### Osteogenic differentiation determination

Quantitative determination of ALP activity of DPSCs under the influence of the different bone substitute materials was performed after a cultivation period of 7 and 14 days using the ALP activity assay kit (Cat. No. PK-CA577-K412, PromoKine, Germany), which uses p-nitrophenyl phosphate as a phosphatase substrate. The absorbance of the samples was measured by spectrophotometric reader at 405 nm. The determination of total protein concentration was performed using a micro-BCA assay kit (Cat. No. 23235, Thermo Fisher, USA), and the ALP activity was finally normalized to the corresponding total protein (OD/mg total protein). DPSCs culture without bone substitute served as control.

As described in a previous study [38], alkaline phosphatase (ALP) staining was used to evaluate the early- and late- phase osteogenic differentiation in various groups after 7 (five images per group) and 14 days (five images per group). DPSCs culture without bone substitute served as control. The culture medium was aspirated, and the cells were gently washed with PBS buffer twice. Fixing solution was then added to the cells, which were incubated at room temperature for 5 min. The fixing solution was then aspirated, and freshly prepared ALP staining solution (cat. no. GR3358974-1, Abcam Co., UK) was added in darkness and incubated at room temperature for 15 min. Finally, ALP-positive (ALP +) cells (purple) were observed via optical microscopy. For the analysis of the ALP + area, the images were converted to 8-bit gray scale using ImageJ software. The threshold was set using an automated routine, and the immunoreactive area of the entire image was calculated as percentage of pixels. All examinations were conducted via observer blinding.

### Analysis of osteogenic gene expression

The impact of bone graft scaffolds on osteogenic differentiation of DPSCs were examined by quantitative real-time PCR (qRT-PCR). After 7 and 14 days, total RNA was extracted by homogenizing the scaffolds with the cells using a commercial RNA extraction reagent (TRIzol, Invitrogen, USA), and the extracted RNA was transcribed into complementary DNA (cDNA) using an RNA-to-cDNA kit. qRT-PCR analysis was performed using the SYBRTM Green Master Mix Kit (Cat. No. 4385612, Thermo Fisher, USA) to quantify established target genes for osteogenic differentiation, including runt-related transcription factor 2 (*RUNX2*), osteocalcin (*OCN*), and osteopontin (*OPN*) [39]. The results of all gene expressions were normalized to the CT values of the housekeeping gene *GAPDH*. Primer pairs used are listed in Table 2. All assays were performed in triplicate in three independent experiments. DPSCs culture without bone substitute served as control.

**Table 2:** Primer sequences used for qRT-PCR

Gene	Forward primer	Reverse primer	GenBank accession number
*RUNX2*	CGCCTCACAAACAACCACAG	TCACTGCACTGAAGAGGCTG	NM_001278484
*OCN*	CCGTTTAGGGCATGTGTTGC	CCGTCCATACTTTCGAGGCA	NM_013414
*OPN*	CCAGCCAAGGACCAACTACA	GCTGGCAGTGAAGGACTCAT	NM_012881
*GAPDH*	GTGAAGGTCGGAGTGAACGGATT	ACCATGTAGTGGAGGTCAATGAAGG	NM_017008

### Statistical analysis

All data were analyzed with the GraphPad Prism 7.0 program (GraphPad Software, Inc., San Diego, CA, USA). After analysis, the data for parametric statistics were found to have met the D’Agostino–Pearson and Shapiro–Wilk test criteria for normal distribution and passed the Brown–Forsythe test for equal variances. Appropriate results were analyzed by one-way ANOVA, followed by Tukey’s testing. Data for multiple comparison were analyzed using two-way ANOVA, followed by Tukey’s multiple comparison testing. All data in this paper represent the means ± standard deviation (SD). Differences between the groups were considered significant when *p* < 0.05.

## Results

### Bone scaffold morphology

**Fig. 1:**
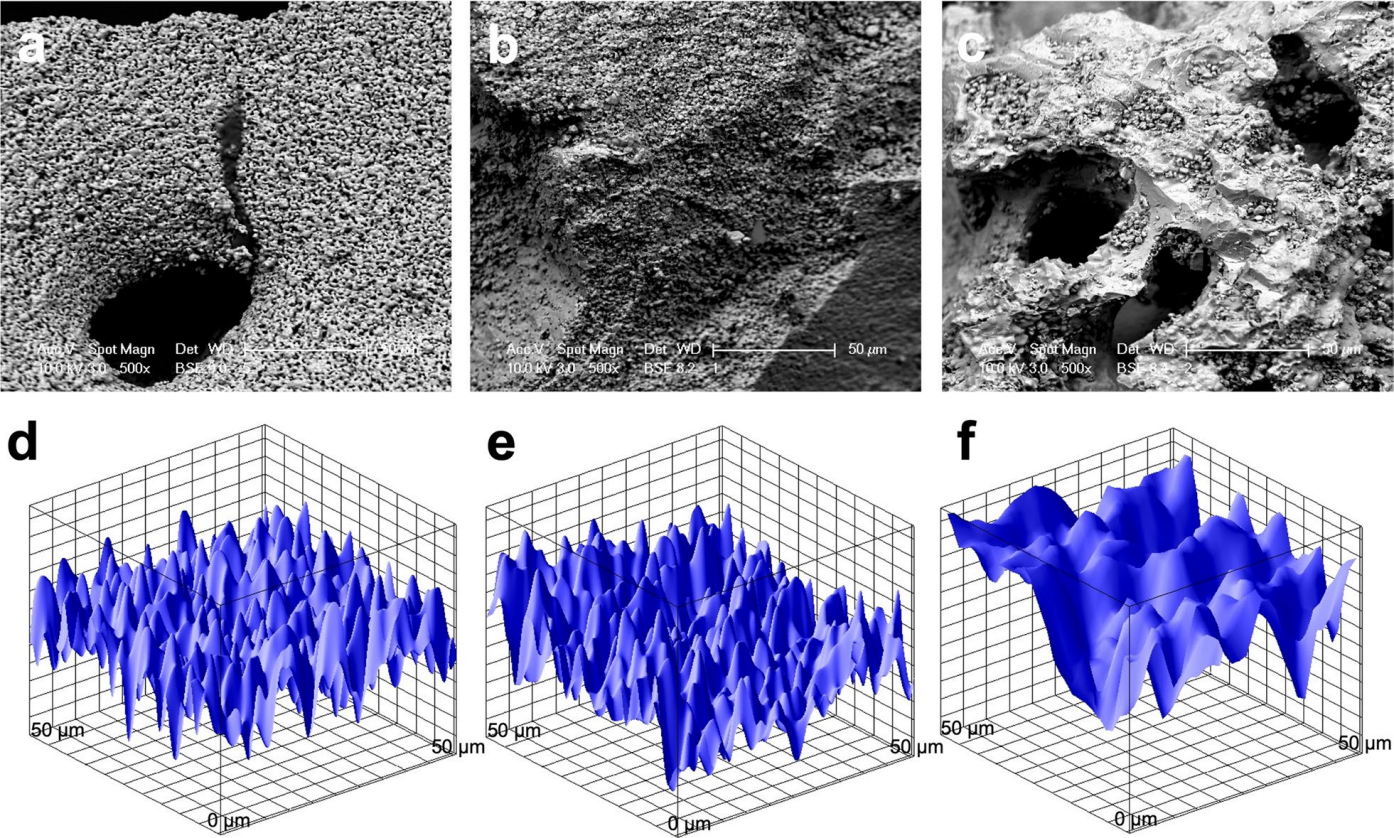
SEM images of unloaded** a** BCP, **b** FDBA and **c** DBBM scaffolds. Surface plots of unloaded **d** BCP, **e** FDBA and **f** DBBM scaffolds. Scale bar = 50 µm

BCP had the most microporosities, with the overall smallest pore sizes of 4.47 ± 1.91 µm. FDBA had fewer microporosities than BCP, with pore sizes of 4.94 ± 1.11 µm, and showed few cracks (24.71 ± 5.7). DBBM had the fewest microporosities (14.88 ± 5.86 µm) and many macroporosities (89 ± 38.88 µm). SEM images and the surface plots of unloaded scaffolds are shown in Fig. 1. While BCP had a homogeneous, bumpy surface, FDBA and DBBM had rough surface textures with cracks.

### Bone substitute concentration and cytotoxicity assay of DPSCs

**Fig. 2:**
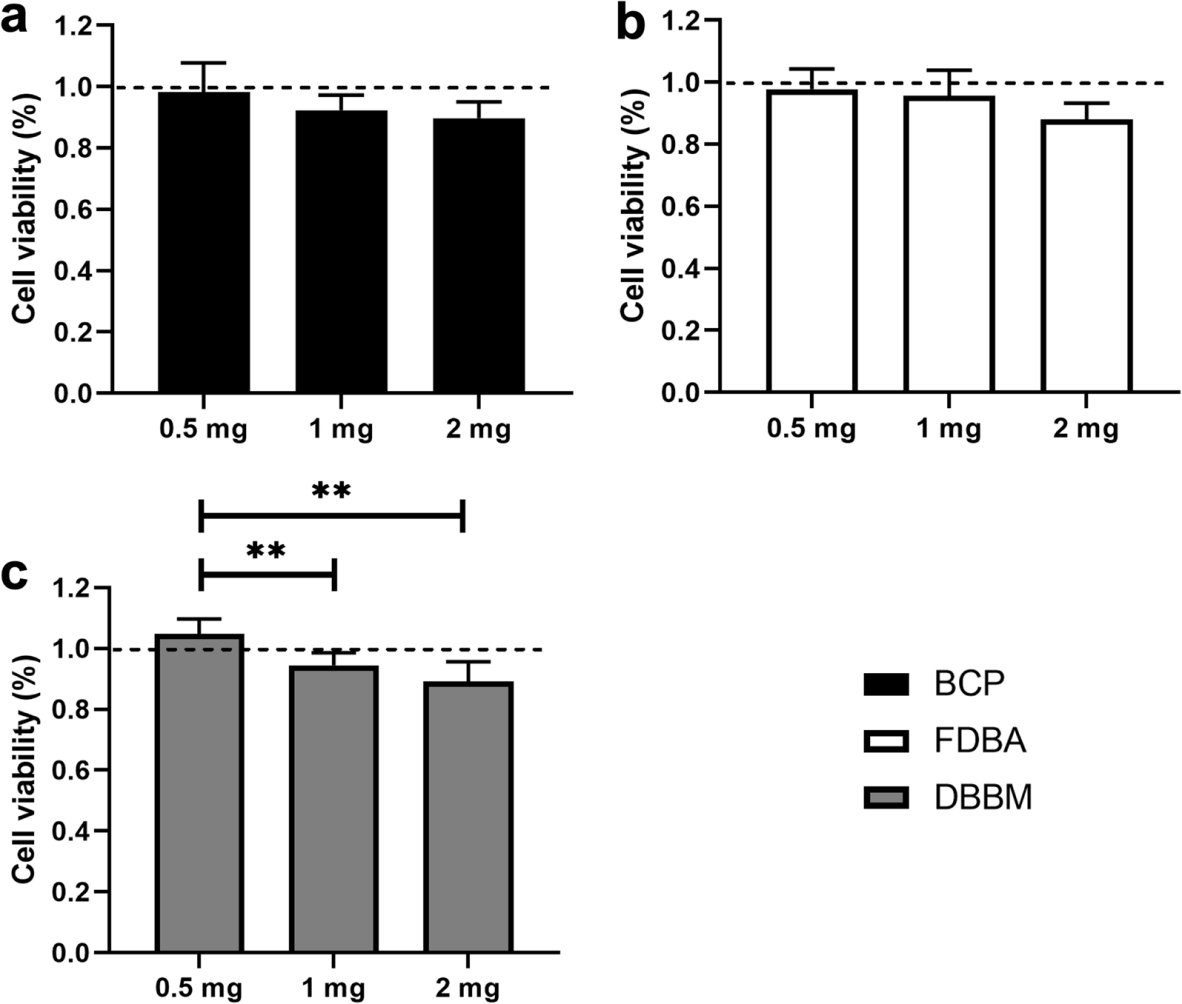
CCK-8 cytotoxicity assay of DPSCs on **a** BCP, **b** FDBA and **c** DBBM bone substitutes with concentrations of 0.5 mg/ml, 1 mg/ml and 2 mg/ml (n=3). CCK-8 enables sensitive colorimetric determinations of live cell counts. ** (*p* < 0.01); *** (*p* < 0.001)

The results of the cytotoxicity tests are shown in Fig. 2. At a concentration of 0.5 mg/ml FDBA, we noted significantly more (*p* < 0.05) viable cells (0.98 ± 0.06) than at a concentration of 2 mg/ml FDBA (0.88 ± 0.05). Likewise, a significantly higher percentage of viable cells was observed at 0.5 mg/ml BCP (0.98 ± 0.1) than at 2 mg/ml BCP (0.9 ± 0.05). A concentration of 0.5 mg/ml DBBM showed the highest overall percentage of viable cells (1.05 ± 0.05), compared to 1 mg/ml (0.95 ± 0.04; *p* < 0.05) and 2 mg/ml (0.89 ± 0.06; *p* < 0.001). For the optimal concentration of bone substitutes in the cell culture media, the cytotoxicity assay proved the highest cell viability to be a concentration of 0.5 mg/ml for all bone substitutes. According to these findings, further experiments were performed with a scaffold concentration of 0.5 mg/ml in the cell culture medium.

### Cell morphology assessment

**Fig. 3:**
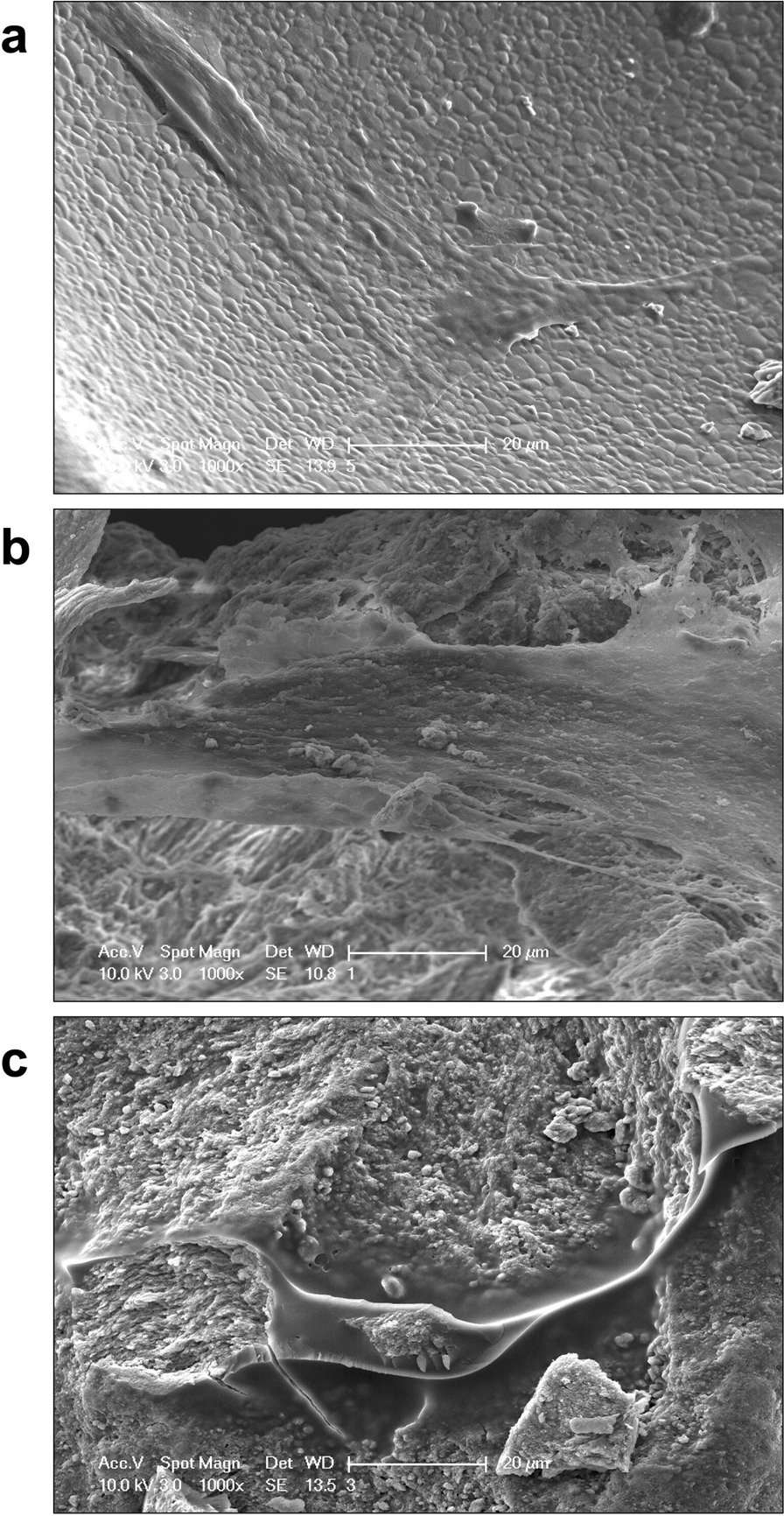
Photographs of scanning electron microscopy of DPSC on **a** BCP, **b** FDBA and **c** DBBM scaffold. The granular surfaces of all scaffolds are covered with singular cell sheets. Scale bar = 20 µm. 1000× Magnification

SEM images of DPSCs after 7 days of culturing are shown in Fig. 3, which illustrates well-attached cells with intercellular connections on the (a) BCP, (b) FDBA, and (c) DBBM scaffolds. On all scaffolds, the cells were very plain, in a wide and elongated shape, which is a typical morphology of healthy cells on scaffolds. Due to more observable intercellular connection, the cell-to-cell connection proved to be stronger among BCP and DBBM, whereas the cell-to-matrix connection was stronger on DBBM. Signs of cell stress due to a humped morphology were not observed in any group.

### Cell proliferation and attachment on bone graft scaffolds

**Fig. 4:**
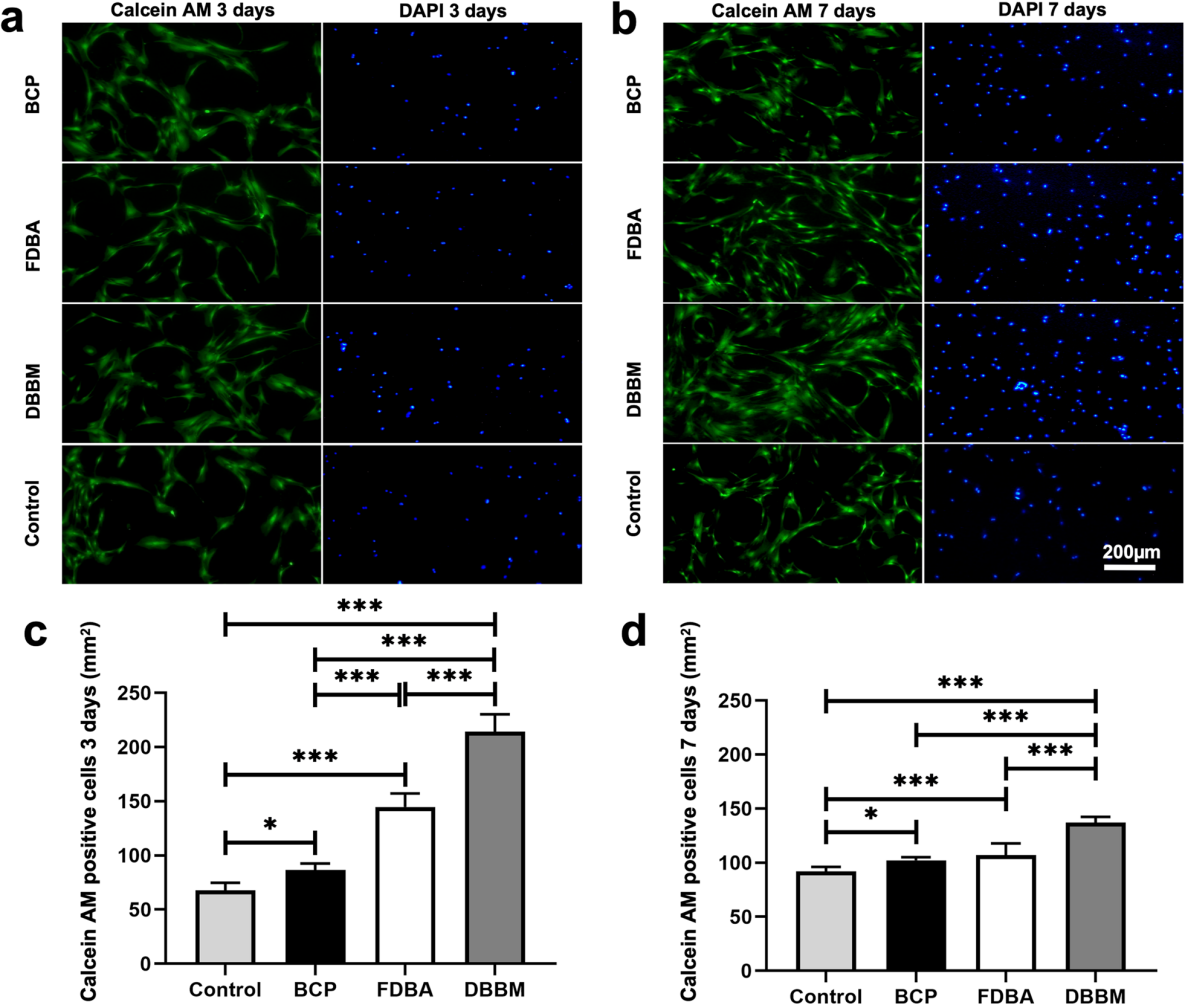
Histological images of calcein AM and of DAPI staining **a** after 3 days and **b** after 7 days. **c** Graphs of calcein AM analysis after 3 days and **d** after 7 days. Stem cells without bone substitute served as control**.** 50× Magnification. *(*p* < 0.05); ***(*p* < 0.001)

**Fig. 5:**
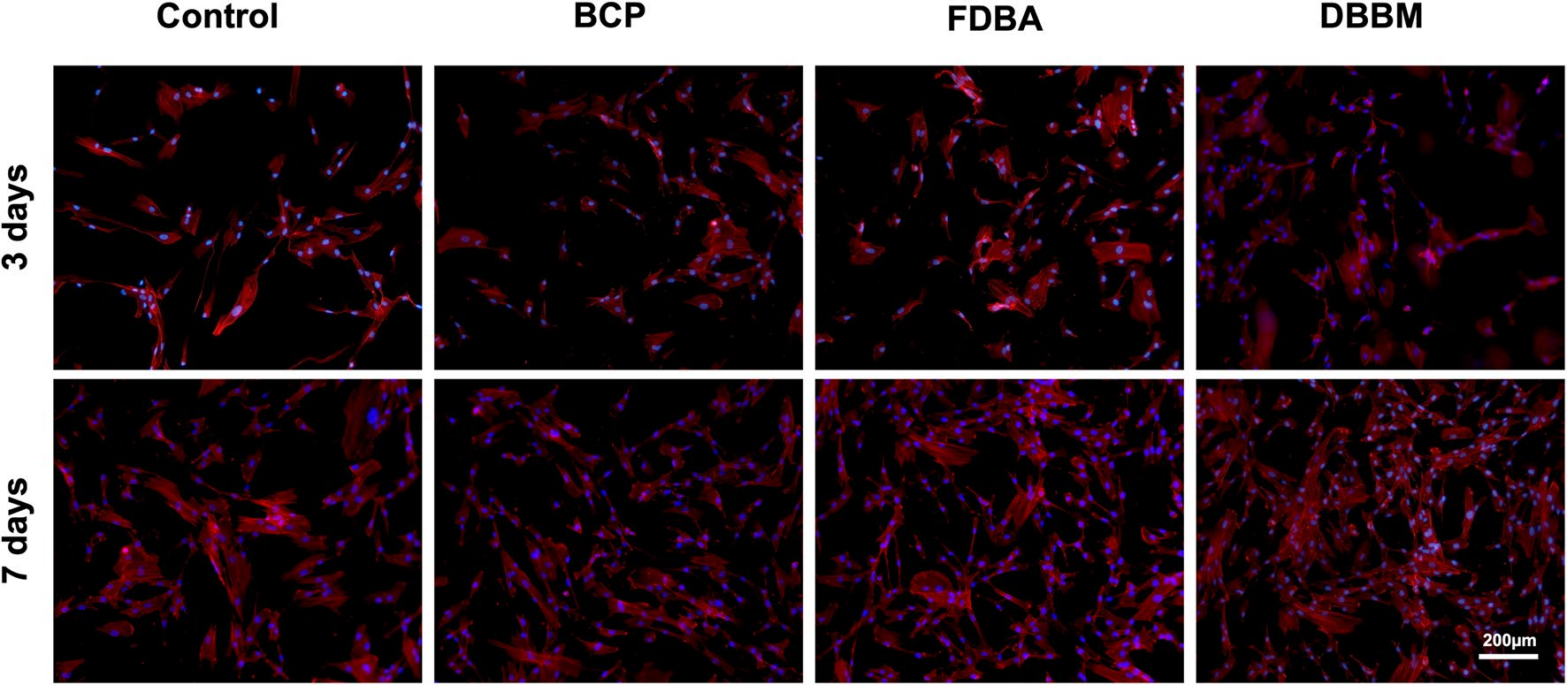
Confocal microscopy images of DPSCs stained with phalloidin and DAPI staining. 100 × Magnification. Stem cells without bone substitute served as control

The results of calcein AM staining are shown in Fig. 4. The DBBM scaffold showed significantly higher proliferation and adhesion of DPSC after 3 days (137 ± 5.37; *p* < 0.001) and after 7 days (214.25 ± 16.17; *p* < 0.001), respectively. The amount of calcein AM–positive cells of BCP (86.25 ± 6.32) showed the lowest proliferation and attachment of DPSC than FDBA (144.75 ± 12.49; *p* < 0.001) after 7 days. Figure 5 shows the phalloidin staining. Consistent with the results of calcein AM staining, the DBBM scaffold resulted in a relatively dense cytoskeletal network layer with a high number of well-proliferating DPSCs. In contrast, in the BCP and FDBA scaffolds, the staining showed lower cytoskeletal proportions and fewer proliferated DPSCs.

### Osteogenic differentiation determination

**Fig. 6:**
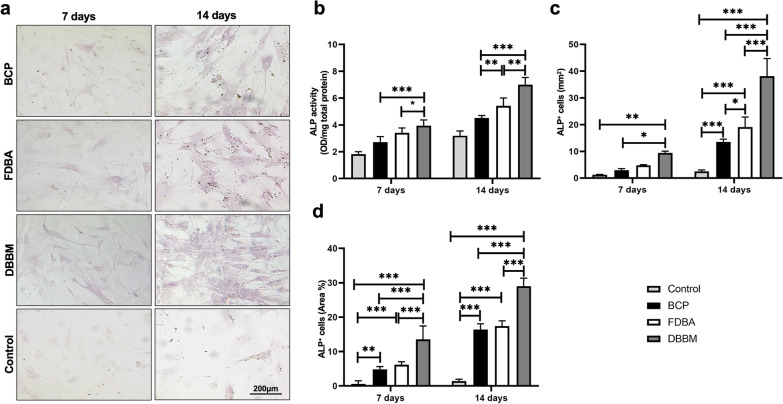
**a** Histological images (100× Magnification) and graphical illustrations of **b** ALP activity after 7 days and 14 days. *(*p* < 0.05); **(*p* < 0.01); ***(*p* < 0.001). **c** Graphs of ALP^+^ cells and **d** ALP^+^ area at osteogenic differentiation after 7 and 14 days of DPSC cell culture. *(*p* < 0.05); **(*p* < 0.002); ***(*p* < 0.001). Stem cells without bone substitute served as control

Figure 6 shows (a) illustrations and the results of (b) ALP activity and (c–d) ALP staining after 7 and 14 days. ALP activity is significantly increased in all three scaffolds compared to control at both time points. At day 14, the significant highest ALP activity was measured on the DBBM scaffold (7.0 ± 0.53) compared to the BCP (4.52 ± 0.19) (*p* < 0.001) and FDBA (5.42 ± 0.59) (*p* < 0.01) scaffolds, respectively. DPSCs with DBBM showed the most significantly osteogenically differentiated cells (15 ± 2.45) compared to FDBA (6.5 ± 0.85; *p* < 0.002) and BCP (5 ± 0.82; *p* < 0.001). The area of positive ALP staining (9.44 ± 0.62%; *p* < 0.001) was the highest in DPSC and DBBM. With an area of 2.91 ± 0.65%, DCPS with BCP showed the lowest osteogenically differentiated cell surface compared to DBBM and FDBA (4.75 ± 0.26%; *p* < 0.002). After 14 days, the DPSCs on the DBBM scaffold showed highly significantly increased ALP expression (*p* < 0.001) as well as a significantly larger area of ALP positive cells (*p* < 0.001) compared to the other materials.

### Analysis of osteogenic gene expression

**Fig. 7:**
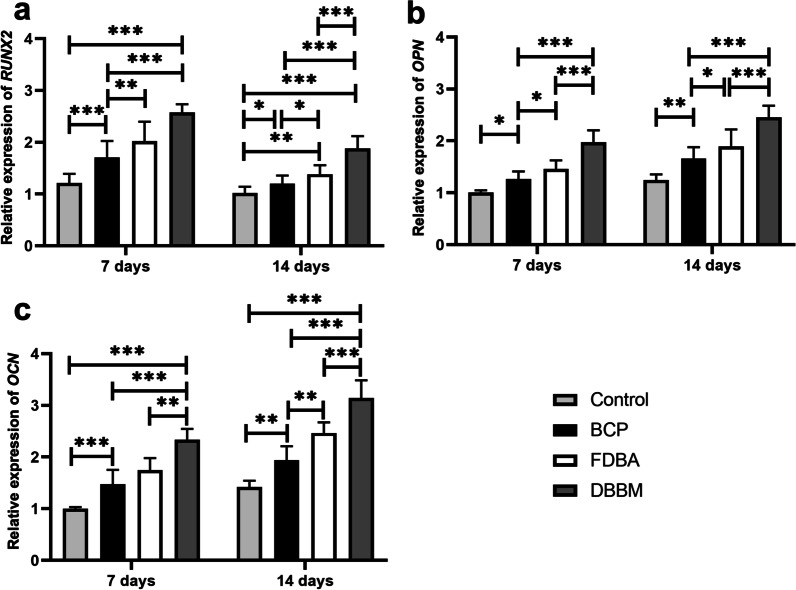
Graphical illustrations of qRT-PCR of **a** *RUNX2*, **b** *OPN* and **c** *OCN* after 7 and 14 days. *(*p* < 0.05); **(*p* < 0.001); ***(*p* < 0.001)

Figure 7 shows that DPSCs expressed the early osteogenic marker gene *RUNX2* at a significantly higher level on the DBBM scaffold (2.57 ± 1.6) than on BCP (1.71 ± 0.32; *p* < 0.001) at day 7. At 14 days, total *RUNX2* expression is decreased compared to levels at 7 days, but remains significantly higher in DBBM (1.88 ± 0.24) than in all other groups tested (*p* < 0.001). In addition, after 14 days, the expression of mature osteogenic genes (*OCN* and *OPN*) was significantly increased on the tested BCP, FDBA, and DBBM scaffolds compared with the control, with the highest *OCN* and *OPN* expression on the DBBM scaffolds, with (3.14 ± 0.34; *p* < 0.001) and (2.45 ± 0.23; *p* < 0.001), respectively.

## Discussion

The aim of this study was to analyze the osteogenic capability of DPSCs on three common bone scaffolds (BCP, FDBA, and DBBM) according to proliferation, attachment, and differentiation. In this in vitro study, all three bone substitutes demonstrated the ability to carry DPSCs.

To the authors’ knowledge, this study is the first in which the physical properties and cytotoxicity of the three bone graft substitutes maxresorb®, maxgraft®, and cerabone® were investigated in relation to their concentrations on DPSC. The influence on the carrier response of bone graft substitutes depends on the physical properties of the surface texture, the expression of micropores, the pore sizes, [23, 34] and the presence of cracks. In this study, BCP showed the most microporosities, with the overall smallest pore sizes (4.47 ± 1.91 µm). These findings were also noted by Motamedian et al*.* [23], who described the highest percentage of microporosities (with dimensions of 3–17 µm) in a comparable alloplastic preparation, which consisted predominantly of ß-TCP. Bernhardt et al. [40] and Handschel et al. [41] described in their in vitro experiments that the surface properties of the alloplastic bone graft substitutes allow a flat attachment and long stretching of the cells, which is in agreement with the findings of this study. In contrast, mesenchymal stem cells have been described to grow into the pores [40], but this could not be confirmed in this study or in the study by Motamedian et al*.* [23].

The micropore sizes of FDBA were 4.94 ± 1.11 µm in the present study. A comparable FDBA preparation has been reported in the literature with micropore sizes of 2.5–10 µm [23]. A high surface roughness compared to a smooth surface has been described with a higher adherence of mesenchymal stem cells in FDBA bone graft substitutes [23, 42]. According to findings in the literature, a higher number of adherent cells was evident on rough edges and not on smooth soft surfaces. DBBM was found to have the smallest microporosities (14.88 ± 5.86 µm) in the present study. Consistently with stem cells in FDBA, mesenchymal stem cells accumulated more in areas of increased surface roughness on the DBBM, and DPSCs further penetrated into macropores and showed pronounced adherence to edges of the bone graft substitute (Fig. 3).

The percentages of viable cells on BCP (0.98 ± 0.1), FDBA (0.98 ± 0.06), and DBBM (1.05 ± 0.05) have been shown to have the lowest cytotoxicity at 0.5 mg/ml on DPSCs. Our study has clearly demonstrated that the amount of bone graft substitutes had a cytotoxic effect on DPSCs. Interestingly, few studies have described the concentrations of bone graft substitutes [23] or used them in different concentrations of 16.5 mg/well [43] or 30 mg/ml [44] without prior determination of the optimal concentration of each bone graft substitute. Prior cytotoxicity determination for bone substitute materials should be pursued, and the findings of these earlier studies should be interpreted critically, since they did not consider a significant impact on stem cell growth by the amount of bone materials present.

Xenogeneic DBBM showed significantly higher proliferation and attachment of DPSCs after 3 days (137 ± 5.37) and after 7 days (214.25 ± 16.17), respectively, than the BCP and FDBA bone substitutes examined in the present study. The alloplastic bone graft substitute even showed the lowest values (86.25 ± 6.32) in this study, which contradicts the findings of Motamedi et al*.* [23] In the present study, the alloplastic bone graft substitute showed superior values due to proliferation and attachment compared with the allogeneic and xenogeneic grafts. In contrast, the alloplastic bone graft substitute Motamedi et al*.* used was composed of 100% β-TCP, whereas the BCP used in the present study was composed of 60% hydroxyapatite and only 40% β-TCP. Furthermore, the alloplastic and xenogeneic bone graft substitutes in particular were found to differ in their reprocessing, resulting in varying chemical compositions. This is the first time that a study has analyzed the influence of the bone graft substitutes maxresorb®, maxgraft®, and cerabone® on the proliferation of DPSCs.

Demineralization during the preparation of allogeneic and xenogeneic bone substitutes exposes bone-inducing proteins in the bone matrix. The osteoinductive effect is particularly attributed to the presence of the bone morphogenic protein (BMP), in addition to other proteins [45]. Therefore, synthetic ceramics such as BCP do not possess osteoinductive properties [45]. The current widespread use of FDBA and DBBM is based on this osteoinductive ability of the bone substitutes [45]. In accordance with these findings, lower osteogenic differentiation of DPSCs was detected in the BCP group. Compared to the other three bone graft substitutes, the level of ALP activity (with 7.0 ± 0.53), and ALP + DPSCs (with 15 ± 2.45 cells) showed the most osteogenic differentiation in DBBM. In accordance with the increased expression level of *RUNX2* in FDBA and DBBM in this study, Toth et al. described an isolated osteoinductive effect of BMP in their cell culture study with human DPSCs from wisdom teeth, which resulted in significantly increased *RUNX2* levels. The increased expression of the osteogenesis-related genes *RUNX2, OPN*, and *OCN* of DPSCs in the FDBA and DBBM groups, could be explained by the presence of intra-bony proteins, such as BMP, which are components of these bone substitutes. Further studies are needed to validate these findings.

The DBBM in this study consisted of 100% hydroxyapatite. Hydroxyapatite has already been shown in numerous other studies to induce enhanced osteoblastic differentiation of DPSCs [8, 46] as well as promoting the induction of osteoblastic genes, such as ALP [8] or RANK ligand (RANKL), of MSCs [47]. Another influence on the osteogenic differentiation of DPSCs described in the literature is cell culture density. In contrast to thin cell culture conditions of 5 × 10^3^ DPSC cells/cm^2^, a particularly high degree of osteogenic differentiation has been described for dense cell culture conditions of 1 × 10^5^ cells/cm^2^ [18]. In the present study, an established density of 2 × 10^4^ cells/cm^2^ was used. Future studies should investigate this osteogenic effect by using a higher stem cell density of 1 × 10^5^ cells/cm^2^ with the bone graft substitutes BCP, FDBA, and DBBM at a concentration of 0.5 mg/ml.

The limitations of the current study are the circumstances of any in vitro experiment, which cannot reflect the factors and effects of recipient beds, tissue mechanics, different tissue environments, inflammatory processes, and the interactions of different cell types. Nevertheless, in vitro studies are useful for analyzing the specific findings of the interaction of stem cells with different materials and for avoiding animal experiments.

## Conclusion

On the basis of the in vitro results of scanning electron microscopy (SEM) investigation, cell viability assays, proliferation and attachment staining, and analysis of osteogenic gene expression, we may conclude that this study has shown the suitability of maxresorb®, maxgraft®, and cerabone® for colonization with human dental pulp stem cells (DPSCs) for tissue engineering applications, especially for the treatment of maxillomandibular bone defects. The deproteinized bovine bone mineral (DBBM) cerabone® in particular showed the most favorable properties in terms of proliferation, attachment, and osteogenic differentiation. Further experiments will be required to compare these findings in vivo.

## Data Availability

The datasets used and/or analyzed during the current study available from the corresponding author on reasonable request.
